# The Global Assessment of Oilseed Brassica Crop Species Yield, Yield Stability and the Underlying Genetics

**DOI:** 10.3390/plants11202740

**Published:** 2022-10-17

**Authors:** Jaco D. Zandberg, Cassandria T. Fernandez, Monica F. Danilevicz, William J. W. Thomas, David Edwards, Jacqueline Batley

**Affiliations:** 1 School of Biological Sciences, University of Western Australia, Perth, WA 6009, Australia; jaco.zandberg@research.uwa.edu.au (J.D.Z.); cassandria.tayfernandez@research.uwa.edu.au (C.T.F.); monica.danilevicz@research.uwa.edu.au (M.F.D.); william.thomas@research.uwa.edu.au (W.J.W.T.); 2 Center for Applied Bioinformatics, School of Biological Sciences, University of Western Australia, Perth, WA 6009, Australia; dave.edwards@uwa.edu.au

**Keywords:** *Brassica*, oilseed, mustard, yield, security, genotype and phenotype

## Abstract

The global demand for oilseeds is increasing along with the human population. The family of Brassicaceae crops are no exception, typically harvested as a valuable source of oil, rich in beneficial molecules important for human health. The global capacity for improving *Brassica* yield has steadily risen over the last 50 years, with the major crop *Brassica napus* (rapeseed, canola) production increasing to ~72 Gt in 2020. In contrast, the production of *Brassica* mustard crops has fluctuated, rarely improving in farming efficiency. The drastic increase in global yield of *B. napus* is largely due to the demand for a stable source of cooking oil. Furthermore, with the adoption of highly efficient farming techniques, yield enhancement programs, breeding programs, the integration of high-throughput phenotyping technology and establishing the underlying genetics, *B. napus* yields have increased by >450 fold since 1978. Yield stability has been improved with new management strategies targeting diseases and pests, as well as by understanding the complex interaction of environment, phenotype and genotype. This review assesses the global yield and yield stability of agriculturally important oilseed *Brassica* species and discusses how contemporary farming and genetic techniques have driven improvements.

## 1. Introduction

**Figure 1:**
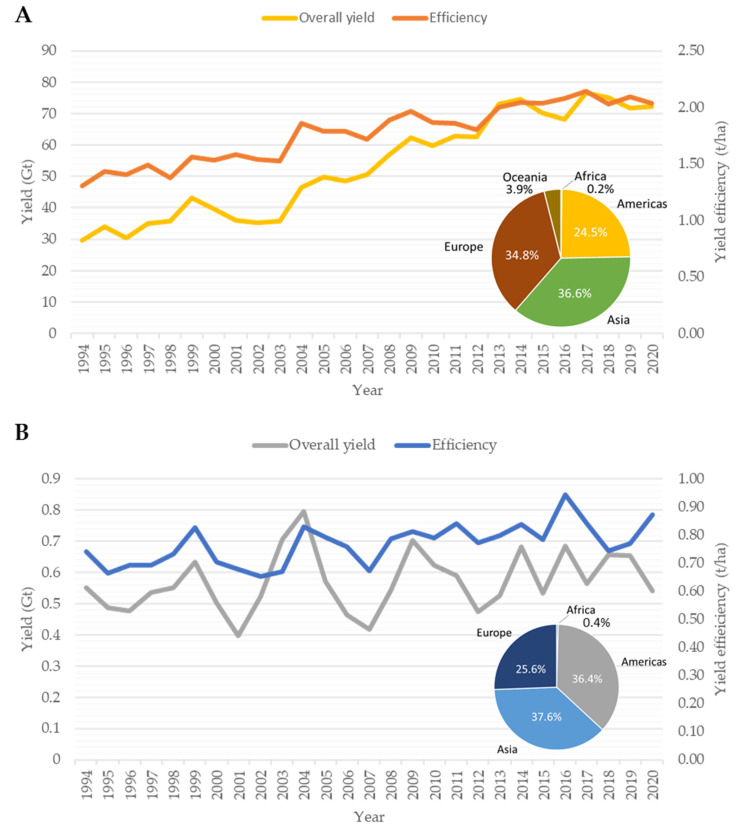
Global yield, land usage and national contribution summary of major oilseed and mustard *Brassica* crops. (**A**) *Brassica napus* (**B**) *Brassica carinata*, *Brassica nigra* and *Brassica alba*.

The Brassicaceae family consists of 4636 accepted taxa, divided into 340 genera and 52 tribes [1,2]. *Brassica* is considered the most important as it contains many of the economically important crops such as oilseeds and condiment varieties, or cruciferous vegetables [3]. Despite the extreme phenotypic variation between *Brassica* spp. [3,4], only a select few are commonly cultivated. For example, the mustard crops *Brassica carinata* (Ethiopian mustard), *B. nigra* (black mustard), *B. alba* (white mustard) and *B.juncea* (brown mustard) are typically grown for the production of condiments due to the taste of their oils ranging from sweet to spicy. Whereas *B. napus* (rapeseed, oilseed rape, canola) is grown as a major source of oilseed for the production of edible vegetable oil [5], as the derived oil has healthy characteristics such as having less than 2% erucic acid and less than 30 µmol g-1 of aliphatic glucosinolates in the meal [6]. Spurred on by the constant need for improvements, breeding programs have developed new varieties with altered oilseed characteristics to fit certain niches. This has in turn provided farmers with a suite of cultivars to choose from, each yielding seeds with different health benefits or industrial uses, for example oilseed with no erucic acid, low levels of gluconisolates, high levels of antioxidants (phenolic compounds), varying vitamin content (C, B9 and K) and the inclusion/exclusion of lutein [7]. Furthermore, with the development of *B. napus* hybrid lines, such as the dual-purpose winter-hybrid and high stability oil varieties, the annual global production has steadily increased each year [8]. In 1979, the global *B. napus* crop yield was recorded to be ~154.2 Mt [9], in 1994, the global yield increased to 34.1 Gt, with no commercially available hybrid varieties. In 2020, the global yield was recorded to be 72.37 Gt with the majority being hybrid varieties (Figure 1A) [10]. In contrast to *B. napus*, the global yield of *Brassica* mustard crops has been highly variable (Figure 1B). In 1994, the global average yield of mustards was 552.3 Mt, with the following years showing steady increase, until 1999, where global yield plummeted. Thereafter, global yield peaked intermittently at 2004 (796.7 Mt), 2009 (704.1 Mt), 2014 (682.2 Mt) and 2016 (685.9 Mt) (Figure 1B). Over the last 26 years, *Brassica* mustard crop yield has decreased by ~2.1% indicating the crop may be grown in non-optimal environments or in volatile socio-economic climates [10].

**Figure 2:**
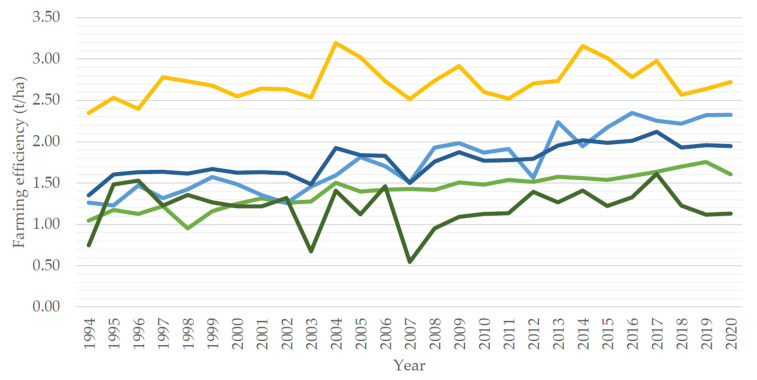
Global and national farming efficiency of *B. napus* oilseed production over 26 years. **Yellow**—Europe, **Light blue**—Canada, **Dark blue**—Global, **Light green**—Asia, **Dark green**—Australia.

The global production of *Brassica* mustard crops is expected to increase due to national renewable energy directives being established. For example, *B. napus* was the only oilseed crop grown in Brazil for decades due to its ability to grow well under the tropical climate, however, with the national drive for production of biodiesel, several *B. juncea* and *B. rapa* varieties were found to be better alternatives due to their oilseed properties (such as higher erucic acid content (22:1) [11]. In contrast, *B. napus* and *B. carinata* are prioritized as sources of oil for biofuel in the US, UK and the EU [12,13,14] as central Europe is well suited for growing winter varieties of *B. napus* [8]. For example, in a ten-year average (2010–2020), the EU achieved the highest farming efficiency (yield/area used) of ~2.76 t/ha (Figure 2) globally, whereas Canada and Asia averaged ~2.11 t/ha and 1.59 t/ha, respectively. The main contributor to the difference is climate; the EU has an extended growing period (particularly long day photoperiods) and growing seasons compared to Asia and Canada [15,16].

National socio-economic position and climate are major drivers for yield stability. For example, in 2017, the EU shifted their focus from growing *B. napus* and instead began relying on imports from Australia and Canada, after both were able to meet the strict EU Renewable Energy Directive greenhouse gas criterion [17]. This led to a significant decrease in production (2.98–2.54 t/ha). However, both drivers can be overcome through by developing new and improved crop management/enhancement programs. China’s production of *B. napus* maintained a slow and steady increase between 2005 and 2015, thereafter, with the years following (2015–2020) seeing a greater increase in farming efficiency, resulting in the national best farming efficiency of 1.753 t/ha (Figure 2) [10]. This shift indicates a concerted effort towards improving yield from given area farmed, rather than relying on increasing the area farmed for increasing yield due to space limitations.

Several other nations have also begun investing in oilseed enhancement programs. For example, the Canola Council of Canada has projected an overall increase in *B. napus* yield from ~21 Mt in 2020 to ~25 Mt in 2025 as per their enhancement programs to meet global demand [18,19]. Furthermore, the Australian Field Applied Research (AFAR) initiative showed that Australia is capable of producing *B. napus* yields in excess of 6 t/ha [20] nearly doubling Europe’s efficiency. This is an amazing feat, as Australia’s production of oilseed crop has consistently been the lowest globally since 2006 due to the extreme climate, such as a short growing season, high temperatures and low rainfall. Climate is a significant constraint and often dictates which crop can be grown. For example, the preferred oilseed crop in India is *B. juncea* due to consistent rainfall, whereas *B. napus* is often selected for its drought tolerance [15]. The yield of *B. juncea* in the region has increased from 0.76 Mt in 1950, to 7.98 Mt in 2020, however, it still does not meet the domestic demand for edible oils.

Yield and yield stability of *Brassica* oilseed and mustard crops currently relies on the optimization of agronomic practices by enhancement programs, creating new varieties through breeding programs and improving management programs to prevent disease and pest incursions. All these programs have gained significant momentum with the integration of molecular resources such as characterization of germplasm, breeding pedigrees and identification of trait-associated loci in breeding programs. Karim et al. 2014., showed that high yielding, short duration *B. napus* lines with up to 4.6 times higher yields can be rapidly created when crossed with hybrid *Brassica rapa* and/or *Brassica oleracea* [16]. However, targeting a particular phenotypic trait such as yield, or oilseed content is not always straightforward. Crop genotypes do not always correlate with crop phenotype. For example, quantitative disease resistance in *B. napus* has been shown to improve yield stability by providing enhanced immunity against several pathogens such as *Leptosphearia maculans* (blackleg), *Sclerotinia sclerotium* (Sclerotinia stem rot) and *Plasmodiophora brassicae* (clubroot), however the underlying genes have yet to be identified due to the multi-genic nature of quantitative resistance, despite substantial genomic resources being available such as reference genomes or pangenomes [5,21,22,23,24]. Furthermore, phenotypic traits often dictate regional viability as the main influence of yield security are stressors such as biotic (disease and pests) and abiotic (temperature, salinity, pH, heavy metals and hydration) stress. More emphasis has been applied to targeting biotic stress resistance phenotypes as unmanaged biotic stress leads to yield loss. Annually, *B. napus* yield is affected by 10–20% loss in UK and Canada and up to 90% in Australia, due to *L. maculans*, the causal agent of blackleg [25,26,27]. As such, to aid in improving *Brassica* crop yield and yield security, this review will assess current farming and disease management programs; introduce the gap between phenotype and genotype; discuss the underlying genetics of yield; and discuss studies that have adopted molecular technology to develop new and improved *Brassica* crops.

### Enhancing Crop Yield and Yield Stability with Farming Techniques and Management Plans

Crop yield and yield stability has been influenced by human intervention for centuries. However, in the future, crop yield and stability will become more affected by the consequences of human intervention. Ray et al. 2019., performed an extensive study of ten global crops, including *B. napus*, relating observed yield to observed weather from 1974–2013 and found that climate change may have already affected canola production. Since the 1970s, the growing season temperature has increased by 1.2 °C and this change has likely affected canola production globally [28]. As a result, the study found that the mean production of canola in western and eastern Europe decreased by 11.4%, North and Central America decreased by 0.4%, whereas in northern Europe, Asia and Oceania it increased by 3.1%, 5.9% and 0.6%, respectively. However, the declining trend of *B. napus* production does not continue past 2013, with the following years (2013–2020) of global production increasing both in yield (averaging ~72 Gt) (Figure 1A) and efficiency (averaging ~2.06 t/ha, Figure 2) [10]. This is most likely due to the rapidly developing molecular resources available (discussed later in Underlying genetics dictate crop yield), integrated farming strategies and adaptive management programs. Several studies have shown through integrating molecular resources and farming techniques, crop yield, yield stability and yield quality can be secured [29,30,31,32,33].

Environmental conditions are the greatest influencers of biomass accumulation, yield during the growth period [6] and may influence plant immunity [34,35,36,37,38,39]. The critical period is the phase of growth in which abiotic stresses have the greatest influence on yield [40]. Kirkegaard et al. 2018., showed that the critical period for canola is between 100 °Cd to 400 °Cd after the start of flowering. The critical period for the other *Brassica* oilseed crops is yet to be established. The second major period is the seed-filling period, also known as grain-fill. The environmental conditions during this time have been found to influence seed size and oil content [6]. Furthermore, the harvested yield is dictated by monitoring the seed branches and meristem for colour change; Graham et al. 2017., found that once 60–80% of all pod branches have changed colour the canola should be harvested to achieve maximum yield [41]. Along with time of harvesting, plant density also dictates maximum yield and harvestability [42,43,44,45,46,47,48]. The angle of the lowest branches decreases as row spacing increases for *B. napus*. As such, not only does high density improve total crop yield, it also improves the actual yield captured through mechanical harvesting [43]. In contrast, in *B. carinata*, row spacing affected seed and oil yield, branch production and the number of pods per plant [46], implying plant density must be carefully evaluated for climate and oilseed crop used.

Plant density differs by climate. In China, the optimum density for *B. napus* is 58.5 × 10^4^ plants/ha [45,49]; in Europe, the optimum density is ~80–150 × 10^4^ plants/ha [50,51]; in Canada, the optimum density is ~50–80 × 10^4^ plants/ha [51]; and in Australia (Western Australia), the optimum density is 25–35 × 10^4^ plants/ha [52]. Furthermore, Ming et al. 2017, showed that typically with high density plant trials, traits associated with rapid leaf senescence (green leaf index, chlorophyll content and malondialdehyde accumulation) are increased, seed pod traits (pod area index, pod photosynthesis and radiation use efficiency) were increased and root associated traits (root length, root tips, root surface area and root volume) were decreased.

Lastly, yield security of *Brassica* oilseed crops is a major concern. Andert et al. 2021., performed a case study of East-German canola farmers and found that on-field data showed yield instability for winter hybrid varieties, which in turn, may cause farmers to begin growing less canola overall [53]. The concern is borne mainly out of future risk from insects and diseases causing irreparable damage to their seasonal yields. As such, disease management strategies are critical for yield stability. A survey of over 100 growers and agronomists established that the best approach towards disease management involved the integration of farming techniques, managing genetic diversity, and careful use of fungicides [25]. Integrated strategies for improving yield with respect to farming techniques has been reviewed extensively [6,25,51]. In summary, maximum grain yield from *Brassica* oilseed crops is dictated by (1) environmental conditions during the critical period, (2) plant density and (3) disease management strategies; grain quality is dictated by: (1) the environmental conditions during the pod-fill stage and (2) genetic diversity. As such, the complex interaction between genotype, phenotype and the environment together, drive grain yield capacity, quality and stability.

## 2. Bridging the Genetic and Phenotypic Gap

Determining the underlying genetic mechanisms driving crop yield is a complex task, considering the interactions between genetic (G), environmental (E) and GxE forces that may affect crop performance [54,55,56]. Multi-environment studies can be more powerful in detecting smaller effect quantitative trait loci (QTL) controlling complex traits such as yield, allowing for the identification of QTL of pleiotropic effect or QTL that suffer significant effects due to GxE interaction [54,56,57]. For instance, a study using segregating *B. napus* populations showed that 81.5% of the QTL linked to yield were pleiotropic [58]. In *B. napus*, a multi-environment analysis of seed composition traits observed that most QTL suffered from GxE effects, with a major QTL qWIE_N9 associated with seed pigment in over five environments [56]. Another study identified a QTL region on chromosome A09 linked to variation in days to flowering, seed yield and plant height under water limited conditions [58]. However, measuring phenotypic traits of hundreds of plants growing in multiple location breeding trials is costly and time consuming, restricting the number of traits measured and frequency of measurements through crop development.

High-throughput phenotyping (HTP) has emerged as an alternative to manual phenotypic trait measurement, accelerating the process of collecting phenotype information through remote and proximal sensors to measure a variety of plant traits [59,60,61]. HTP sensors can be deployed on stationary platforms at a greenhouse or attached to ground and aerial vehicles in the field. These platforms enable the non-destructive measurement of phenotypic traits, which can be used to identify genetic variants associated with improved crop performance and tolerance to environmental stress [62,63,64]. The use of HTP platforms is less labor-intensive than manually measuring traits and prevents the introduction of biases due to human error or sampling methods [65]. Several international groups have invested in the development of HTP centres that either provide the structure for the collection of HTP datasets or publishes their datasets, such as the Australian Plants Phenomics Facility, International Maize and Wheat Improvement Center (CIMMYT), the Genomes to Fields Initiative and TERRA-REF [66,67]. For instance, the TERRA-REF project collects multiple image types of the plants throughout their development along with environmental data, agronomic information and genomic sequences of hundreds of plant varieties [67]. The in-depth measurements of plant development through HTP offered by TERRA-REF offers a great opportunity to uncover genes linked to crop yield and a better understanding of their combined impact on plant response to environmental conditions. Nonetheless, even less comprehensive HTP datasets offer an advantage over manual phenotype measurement methods as the images collected can be re-analysed and shared with other researchers for further investigation [68].

A wide range of sensors is available for monitoring specific plant traits under field or controlled conditions such as RGB, multispectral and hyperspectral cameras, infrared thermal or LiDAR sensors. Infrared thermography images of the plant canopy contribute to determining crop water stress [69] and assist in identifying QTL associated with stomatal density and canopy temperature in *Setaria* [70] https://sciwheel.com/work/citation?ids=13155099&pre=&suf=&sa=0, accessed on 4 September 2022. Multispectral and hyperspectral cameras have been widely employed to obtain quantitative measurements of the canopy reflectance throughout plant development, supporting the identification of potassium deficiency and green peach aphid susceptibility [71], classification of fungal infection severity in *B. napus* seeds [72] and seed pod maturity [73]. Besides tracking specific crop traits that would be difficult to track manually, such as measuring canola flower numbers in the field to predict yield [74,75], HTP platforms can substantially expand the temporal resolution and number of traits monitored to identify genetic variation linked to increased crop performance. For instance, an HTP study using weekly images of maize identified candidate genes associated with regulating plant architecture at early development [76]. Similarly, daily HTP measurements of 477 *B. napus* genotypes revealed multiple medium and small effect QTL were associated with early plant growth, most of which were active during short phases of the development [77]. Dynamic QTLs were also observed in a *B. napus* trial that monitored 43 traits across twelve time points, reporting that only 35% of the QTL identified were present on all time points [78]. These studies indicate the need for stage-specific investigations that uncover transient QTL that may play a role in the plant’s early vigour [79].

HTP platforms have the potential to provide ample information regarding the plant phenotype; however, efficient data collection and processing are considered a key constraint for breeding [60,80]. Image data collection and processing protocol must be carefully designed to avoid biases due to lightning and other environmental conditions, mainly if the measured phenotypic traits are based on the tissue spectral reflectance [81,82]. In addition, there is a need to adapt conventional GWAS and GS methods or implement machine/deep learning models to incorporate the rich information from HTP into the genotype selection pipeline [60]. Machine/deep learning currently presents competitive results for predicting phenotypic traits based on genomic data for GS [83], multi-trait and multi-environment prediction [84,85]. Machine/deep learning has the advantage of automatically extracting features from complex data, building an abstract representation of their relationship regarding the prediction target, which is particularly suited to image dataset analysis [86]. Recent studies have applied machine/deep learning to integrate HTP, environmental and genetic data for selecting varieties under field trial. For example, a study on wheat used generalized Poisson regression, a statistical machine learning method, to merge hyperspectral images with environmental and genetic data to predict count phenotypes for GS [87]. Another study used canopy temperature and vegetation indices for GS of wheat, highlighting that adding the HTP features increased the yield prediction by 70% in genomic models [88]. In maize, it was shown that a multimodal deep learning model using multispectral images and genomic data could accurately identify 75% high yield plots at an early developmental stage [89]. The use of HTP also allows monitoring of the phenotypic traits during the plant development under multi-environment trials, helping the identification of varieties better adapted to the changing environmental conditions. Leveraging phenotypic and genomic datasets using the appropriate analysis tools have the potential to accelerate the breeding of higher-yielding *B. napus* varieties. Observing the impact of genomic variation on crop yield can be facilitated by the use of HTP, but a comprehensive database on *B. napus* genetic resources is required to design future resilient crops.

## 3. Underlying Genetics Dictate Plant Yield

In order to improve yield in plants, one must study the underlying genetic mechanisms and related mechanisms, such as plant architecture. Plant architecture (PA) refers to the three-dimensional organisation of the plant, including its morphological characteristics [90,91]. PA modifications are fundamental for high-yield breeding, often linked with a crop’s adaptive ability and yield potential, such as seed oil content [92], silique number, number of seeds per silique and seed weight [93]. Additionally, traits such as plant height, biomass yield and flowering time indirectly influence seed yield [94]. There have been many studies in *B. napus* linking PA with yield. For example, a gene knockout experiment showed that a stop codon mutation in the meristem identity gene APETALA1 affected flower morphology, PA and yield [95]. Additionally, a study in *B. rapa* showed plant yield was significantly correlated with PA-related traits such as main inflorescence length, branch height and branch segment [96]. 

Identifying yield-related traits has been the focus of many genetic studies in *B. napus*. These studies primarily focus on identifying QTL and single nucleotide polymorphisms (SNPs) related to yield and PA. For example, genetic mapping of different genotypes of *B. napus* and genetic mapping identified 190 PA-related genes for 91 unique PA QTL and 2350 yield loci-pairs [96]. In a separate study, a map comprising 7716 DArTseq markers was created from a population of 145 B. napus lines and identified 20 QTL associated with flowering time and grain yield. Twenty-two putative candidate genes for flowering time and grain yield were identified in the QTL region [97]. Raboanatahiry et al. 2018., aligned 972 QTL for seed-yield and yield-related traits in *B. napus* onto one genetic map and identified 92 regions where 198 QTL overlapped. The study showed that the regions identified could be used to select for desired traits. Additionally, 147 candidate genes potentially influencing PA and yield were identified [94]. A SNP array-based genetic map was used to identify 695 QTL for 14 traits, including PA, flowering, silique and other seed-related traits, in *B. juncea*. It also showed that epistasis among loci plays an important role in controlling heterosis in yield of *B. juncea* [98]. 

Many studies in *B. napus* have used GWAS to identify yield-related genetic variants. Using data from 520 *B. napus* accessions, GWAS for seven yield-determining traits; main inflorescence pod number, branch pod number, pod number per plant, seed number per pod, thousand seed weight, main inflorescence yield, and branch yield, identified 128 SNPs and 14 candidate genes for yield improvement [99]. GWAS was also used to identify candidate genes associated with stress tolerance, oil content, seed quality and ecotype improvement for 588 accessions of *B. napus* [100]. Another study used genotyping by sequencing to screen 125 accessions of *B. napus* and identified 85,126 SNPs for GWAS, directly associating 18 SNPs with seed yield and another 61 SNPs with yield-related traits [101]. By understanding the genetic control of PA in crops, more efficient breeding strategies to improve crop yield can be developed [102]. 

A pangenome is the collection of all genes within a species, first coined by Tettlin et al. in 2005 to describe the gene diversity in *Streptococcus agalactiae* [103]. Pangenomes consist of a core genome, containing the sequences shared between all individuals of a species, and the accessory genome (also known as the dispensable or variable genome), which contains the genes that are not found in all individuals. In the last couple of decades, pangenomes have been constructed for various bacteria, fungi, animals and plants, including *B. oleracea* [104], *B. rapa* [105] and *B. napus* [106]. Pangenomes can be assembled in one of three ways: de novo sequencing and comparison; iterative mapping and assembly; and graph-based assembly. These methods have been extensively covered in other reviews, detailing the construction, benefits and disadvantages of each method [107,108].

Unlike single reference genomes, pangenomes allow the capture of sequences affected by structural variation such as presence/absence variation (PAVs) or copy number variations (CNVs), that may affect agronomically important traits such as disease resistance and yield [109,110]. In *B. oleracea*, use of the pangenome has identified multiple genes coding for resistance against other abiotic factors such as drought [111]. Similarly, pangenomes have identified important presence/absence variations (PAVs) in the *Brassica* genus. In *B. oleracea* and *Brassica macrocarpa*, a pangenome was used to identify PAVs associated with disease resistance, secondary metabolites and flowering time [104]. In *B. napus*, the pangenome identified PAVs associated with flowering time, silique length, seed weight and flowering time [106] as well as PAVs and SNPs associated with disease resistance [112,113]. In *B. rapa*, a pangenome was used to identify PAVs associated with flowering time, stress resistance and lignin formation [114]. Pangenomes can be used for further study of QTL and SNPs by acting as detailed references for trait-mapping tools such as GWAS, allowing for improved studies of genetic variation.

Another way to study candidate genes in *Brassicas* is to construct pangenomes based on specific functional traits. Trait pangenomes aim to describe the landscape of genetic variation related to a trait and investigate the impact of genetic variation. Trait-specific pangenomes have been used to study resistance gene analogs in *B. oleracea* and *B. napus* [111,113,115] and have been employed as a reference for resistance gene cloning [116]. Trait pangenomes could help in further dissecting the genetic variability associated with yield under certain conditions, such as low phosphorous deficiency in *B. napus* [117]. Pangenomes have only been introduced to *Brassica* research recently and use of pangenomics in *Brassica* breeding is still in its infancy. However, further understanding of the genetics underlying variation can lead to the development of molecular markers that can be used to predict the location of desirable crop traits and marker-assisted breeding for improvement of crop yield in *Brassicas.*

## 4. Developing New Phenotypes via Genome Editing Technology

Genome editing presents an opportunity to rapidly introduce or manipulate specific traits of interest that may not be present in the existing gene pool of elite crop varieties, or that may be difficult and time consuming to introduce through traditional introgression breeding approaches. The advent of clustered regularly interspaced short palindromic repeats systems associated with Cas enzymes (CRISPR/Cas) has opened the doors for widespread editing in oilseed *Brassicas*, which greatly benefit from sharing homologs of well characterised genes associated with yield in *Arabidopsis*. However, the high gene homology and copy number of the allotetraploid *Brassicas*, such as *B. napus* and *B. juncea*, present a challenge in terms of editing accuracy, in particular single base editing [118], and in gene redundancy via multiple homologs, requiring all genes homologous to the target to be modified to produce a reliable phenotype [119]. Polygenic traits will require a more intricate multiplex approach, targeting more than one locus in a single round of editing. Yield is one such trait that is complex and will likely benefit from a multiplex approach in order to achieve rapid improvement [120]. Although hybrid-CRISPR Cas enzymes have been designed for targeting multiple loci for inactivation or activation [121,122], the technology has yet to be applied in *Brassica*. In *Brassica* oilseeds, yield remains poorly characterised on the molecular level, and knowledge of the genetic mechanisms controlling seed size and oil content is scarce [120]. Hence, many of the traits currently targeted for yield improvement are those that are closely related to yield, or those that are indirectly related, but highly correlated. For example, current research has mainly focused on increasing seed number through changes to silique structure and improving harvestability through a reduction in plant heigh and an increase in plant density [118]. In addition, other traits such as pod shattering resistance [123,124] and flowering time [125], which significantly influence yield, have been investigated.

Not surprisingly, *B. napus* has received the most attention of all the oilseed *Brassicas* when it comes to the use of editing for yield improvement. Yang et al. were the first to examine multilocular siliques in *B. napus* using CRISPR/Cas9 and introduced mutations to CLV pathway genes, resulting in an increased number of multilocular siliques. Multilocular siliques were found to contain more seeds than regular bilocular siliques, and seed weight per silique was increased by 74% in the mutants [126]. A more recent study induced mutations in *BnD*14, a strigolactone receptor, to change architectural traits in B. napus relating to yield [127]. Knockout lines for *BnD*14 displayed dwarfed phenotypes, which are easier to harvest, with prolific branching and nearly 40% more flowers than their WT counterparts. To combat pod shattering, Zaman et al., knocked out homeologs of SHATTERPROOF1/2, a class of genes involved in the pod dehiscence regulatory pathway, creating a transgene-free line that was approximately 10 times more resistant to pod shattering when measured on the pod-shattering resistance index [127]. These examples represent a growing list of studies (list shown in Table 1) that successfully exploit genome editing via CRISPR systems to functionally characterise and manipulate key genes associated with yield or yield-related traits in *B. napus*. Although examples of editing for yield improvement in *B. juncea* are yet to be seen, several potential targets have been identified and characterised, such as genes involved with multilocular silique development [128], and provide a guide for future editing efforts.

As we start to understand the complex mechanisms underpinning yield and identify yield-related genes in oilseed *Brassicas*, exponentially improving their yield through genome editing is only just out of reach. Advances in CRISPR systems in terms of simplicity and accuracy is a driving force propelling CRISPR into the mainstream. Novel CRISPR techniques, such as the recently proposed and highly sophisticated CRISPR-Combo system, which simultaneously enables gene editing and gene activation at multiple loci, will likely spearhead the race to high-yielding genome edited crops [122], as well as DNA-free gene editing technology which has been dubbed ‘the way of true plant editing’ [129,130,131]. These systems will become necessary in future for the development of new and improved crops that have improved yield and yield stability.

**Table 1:** Recent examples of yield-related trait improvement using CRISPR/Cas9 in *Brassica napus*.

Trait	Target Gene	*Arabidopsis* Homolog	Mutant Phenotype	Reference
Flowering time	*BnaSVP*	*Short Vegetative Phase* (*SVP*)	40–50% decrease in time until flowering	[132]
Plant height, internode length and number of branches	*BnD14*	*DWARF14* (*D14*)	34% reduction in plant height, 200% increase in branch number and 37% increase in total flowers per plant	[127]
Plant height and branch angle	*BnaA03.BP*	*BREVIPEDICELLUS* (*BP*)	~16% reduction in plant height and branch angle reduced from 84° to 14°	[133]
Pod shattering resistance	*BnSHP1/BnSHP2* homeologs	*SHATTERPROOF1/2*	~10 times more resistant to pod shattering	[134]
Number of seeds per silique	*BnaEOD3*	*ENHANCER3 OF DA1* (*EOD3*)	Shorter silique length and smaller seeds, but 42% increase of number of seeds per silique	[135]
Pod shattering resistance	*BnJAG.A02, BnJAG.C02, BnJAG.C06, BnJAG.A07, BnJAG.A08 *	*JAGGED (JAG)*	2 times more resistant to pod shattering	[123]
Multilocular silique development	*BnA04.CLV3*, *BnC04.CLV3*, *BnC02.CLV3*	*CLAVATA3* (*CLV3*)	74% increase in seed weight per silique	[126]
Plant height, primary branch number and silique number	*BnaMAX1*	*More Axillary Growth* (*MAX*)	~35% reduction in plant height, 3 times more primary branches and ~65% increase in total silique number	[136]

## 5. Conclusions

Crop yield and yield stability have been the focus of agriculture for thousands of years. However, with realization of climate change and the predicted increase in world population by 2050, concerted efforts are necessary to meet growing demands. As an example of this demand, *B. napus* production has increased from 154.2 Mt in 1979 to 72.37 Gt in 2020 and is now only second to soybean as a source of oilseed. Although primarily grown as a source of vegetable oil or mustard oil, *B. napus* (and to a lesser extent *B. carinata*), *B. juncea* and *B. rapa* have also been identified as excellent sources of biofuel, applying more pressure for studies to maximise yields. The majority of (if not all) studies that are associated with maximising yield involve genetics and/or gene-editing technology as it is a proven and rapid method for yield improvement. The global FE (t/ha) of *B. napus* pre-genetics age (<2003, before the sequencing of the human genome) was 1.48 t/ha, compared to now (2004–2020) which is 1.94 t/ha. Despite the adoption of genetic resources, the global yield of *Brassica* crops is insufficient for current and future demands, in particular mustard crops, however, this is more likely due to yield instability. An investigation of global and national FE clearly shows fluctuations for both *B. napus* and *Brassica* mustard crops in recent years, which can cause repercussions towards the supply chain of these crops, for example, farmers losing confidence in their crops. As such, research studies investigating the influence of environment, phenotype and genotype on crop yield and yield stability are required to produce reliable crops for farmers to meet demands.

Studies have shown that environmental conditions are the greatest influencers of biomass accumulation, furthermore, climate dictates the particular genotypes and phenotypes that can thrive in any one region. High-throughput phenotyping technology allows for the rapid analysis and characterization of new and potential *Brassica* cultivars of their ability to grow well in a region. As an alternative to manual phenotyping, HTP technology accelerates the process of collecting phenotype information through remote and proximal sensors to measure a variety of plant traits all of which are catalogued and integrated. Coupled to deep/machine learning technology, HTP can quickly establish the optimal phenotype (highest yield) for the region. However, HTP is limited by the availability of genomic resources, such as QTL, GWAS and SLAF-seq data associated with plant architecture. As such, genetic studies are critical. However, the underlying genes that are associated with phenotypic variations exhibited by plants are great and nearly impossible to identify by genomics alone. Hence, pangenomic analysis has been developed as a strategy to capture the genetic diversity to bridge the gap between genotype to phenotype. Many *Brassica* crops have pangenomes available such as *B. napus*, *B. rapa* and *B. oleracea*, providing unique insights into DNA polymorphisms, structural variation and patterns for genome duplication. The combination of genomics/pangenomics and phenotypic studies has enhanced the prediction of molecular markers associated with specific crop traits for crop yield and yield stability. Furthermore, like HTP technology leading to improved phenotyping capabilities; gene-editing technology has enhanced genotyping by rapidly characterizing yield-associated candidate genes and creating new genotypes. It is predicted that DNA-free gene-editing technology will lead to the development of non-GMO plant genotypes that can rapidly be produced and adopted by farmers.

The combination of environment, phenotype and genotype dictate yield and yield stability. As such, developing methodologies to understand these factors and their complex interactions are critical for ensuring future demand is met. This review shows how farming practice and management programs, HTP, genomics, pangenomics and gene editing technology have influenced the production and security of *Brassica* oilseed and mustard crops. Only through continued improvement and innovation of these resources can yield increase and be secured.

## Data Availability

This study did not produce any data, however, any data described in ‘Introduction’ can be found in FAOSTAT (https://www.fao.org/faostat/en/#home, accessed on 4 September 2022).
