# Correction: Zandberg et al. The Global Assessment of Oilseed Brassica Crop Species Yield, Yield Stability and the Underlying Genetics. *Plants* 2022, *11*, 2740

**DOI:** 10.3390/plants14020174

**Published:** 2025-01-10

**Authors:** Jaco D. Zandberg, Cassandria T. Fernandez, Monica F. Danilevicz, William J. W. Thomas, David Edwards, Jacqueline Batley

**Affiliations:** 1 School of Biological Sciences, University of Western Australia, Perth, WA 6009, Australia; jaco.zandberg@research.uwa.edu.au (J.D.Z.); cassandria.tayfernandez@research.uwa.edu.au (C.T.F.); monica.danilevicz@research.uwa.edu.au (M.F.D.); william.thomas@research.uwa.edu.au (W.J.W.T.); 2 Center for Applied Bioinformatics, School of Biological Sciences, University of Western Australia, Perth, WA 6009, Australia; dave.edwards@uwa.edu.au

There was an error in the original publication [1]. One of the references included was not on the topic at hand and should be removed. The relevant sentence in second paragraph of Section 4 is corrected as follows: 

Although examples of editing for yield improvement in *B. juncea* are yet to be seen, several potential targets have been identified and characterised, such as genes involved with multilocular silique development [128].

With this correction, the order of some references has been adjusted accordingly. The authors state that the scientific conclusions are unaffected. This correction was approved by the Academic Editor. The original publication has also been updated.
